# Improve-RRBS: a novel tool to correct the 3′ trimming of reduced representation sequencing reads

**DOI:** 10.1093/bioadv/vbae076

**Published:** 2024-05-24

**Authors:** Ábel Fóthi, Hongbo Liu, Katalin Susztak, Tamas Aranyi

**Affiliations:** Institute of Molecular Life Sciences, Research Center for Natural Sciences, HUN-REN, Budapest 1117, Hungary; Department of Molecular Biology, Semmelweis University, Budapest 1094, Hungary; Renal Electrolyte and Hypertension Division, Department of Medicine, University of Pennsylvania, Philadelphia, PA 19104, United States; Penn/CHOP Kidney Innovation Center, University of Pennsylvania, Philadelphia, PA 19104, United States; Department of Genetics, University of Pennsylvania, Philadelphia, PA 19104, United States; Renal Electrolyte and Hypertension Division, Department of Medicine, University of Pennsylvania, Philadelphia, PA 19104, United States; Penn/CHOP Kidney Innovation Center, University of Pennsylvania, Philadelphia, PA 19104, United States; Department of Genetics, University of Pennsylvania, Philadelphia, PA 19104, United States; Institute of Molecular Life Sciences, Research Center for Natural Sciences, HUN-REN, Budapest 1117, Hungary; Department of Molecular Biology, Semmelweis University, Budapest 1094, Hungary

## Abstract

**Motivation:**

Reduced Representation Bisulfite Sequencing (RRBS) is a popular approach to determine DNA methylation of the CpG-rich regions of the genome. However, we observed that false positive differentially methylated sites (DMS) are also identified using the standard computational analysis.

**Results:**

During RRBS library preparation the MspI digested DNA undergo end-repair by a cytosine at the 3′ end of the fragments. After sequencing, Trim Galore cuts these end-repaired nucleotides. However, Trim Galore fails to detect end-repair when it overlaps with the 3′ end of the sequencing reads. We found that these non-trimmed cytosines bias methylation calling, thus, can identify DMS erroneously. To circumvent this problem, we developed improve-RRBS, which efficiently identifies and hides these cytosines from methylation calling with a false positive rate of maximum 0.5%. To test improve-RRBS, we investigated four datasets from four laboratories and two different species. We found non-trimmed 3′ cytosines in all datasets analyzed and as much as >50% of false positive DMS under certain conditions. By applying improve-RRBS, these DMS completely disappeared from all comparisons.

**Availability and implementation:**

Improve-RRBS is a freely available python package https://pypi.org/project/iRRBS/ or https://github.com/fothia/improve-RRBS to be implemented in RRBS pipelines.

## 1 Introduction

DNA methylation is a covalent epigenetic mark essential in physiologic and pathologic processes (Greenberg and Bourc'his 2019). In mammals, DNA methylation almost exclusively targets cytosines of CpG dinucleotides by de novo and maintenance methyltransferases, DNMT3a/3b and DNMT1, respectively. Methylation of promoters and enhancers strongly correlates with gene silencing. Similarly, methylation of repeat sequences plays a crucial role in the maintenance of genome integrity by inhibiting transcriptional and transposase activity of genomic parasites. Heavy DNA methylation also characterizes the body of actively transcribed genes (Jones 2012, Greenberg and Bourc'his, 2019). Gene body methylation might inhibit spurious transcription from intronic promoters, participate in the regulation of miRNA expression and alternative splicing.

Genome-wide DNA methylation varies in time (e.g. during embryonic development and differentiation) and space (between different cell types), leading to cell-type specific methylation and gene expression patterns (Arányi and Páldi 2006, Métivier *et al.* 2008, Jones 2012). Further, environmental factors can induce DNA methylation changes, which also modify gene expression. Genome-wide DNA methylation pattern is also altered in various chronic diseases such as chronic kidney diseases (CKD), diabetic nephropathy, many cancers, hematopoietic malignancies, and neurodevelopmental diseases (Lyst and Bird 2015, Pagliaroli *et al.* 2016, Jeong *et al.* 2018, Gluck *et al.* 2019, Skvortsova *et al.* 2019, Tulstrup *et al.* 2021, Beard *et al.* 2023). These methylation changes can be diagnostic, prognostic, and used as biomarkers, and even some of these diseases are treated by methyltransferase inhibitors. Therefore, the analysis of DNA methylation is of great interest.

Several methods, based on Southern blot, restriction enzymes, antibody detection, mass spectrometry, and chemical modification were developed during the last decades to investigate DNA methylation (Grunau *et al.* 2001, Zilberman and Henikoff 2007, Kurdyukov and Bullock 2016, Karemaker and Vermeulen 2018, Vető *et al.* 2018). The gold standard has become the bisulfite treatment-based method (Grunau *et al.* 2001). This chemical treatment of the DNA distinguishes between methylated and unmethylated cytosines, allowing their detection by a multitude of techniques coupled to the bisulfite treatment. The two most frequently used detection techniques are the hybridization and the sequencing methods. Hybridization-based techniques are mainly restricted to human genomic DNA samples (Bibikova *et al.* 2011, Zhou *et al.* 2017) and very recently to mouse (Zhou *et al.* 2022), while sequencing can be applied to any sample type and be either targeted or genome-wide.

**Figure 1.**
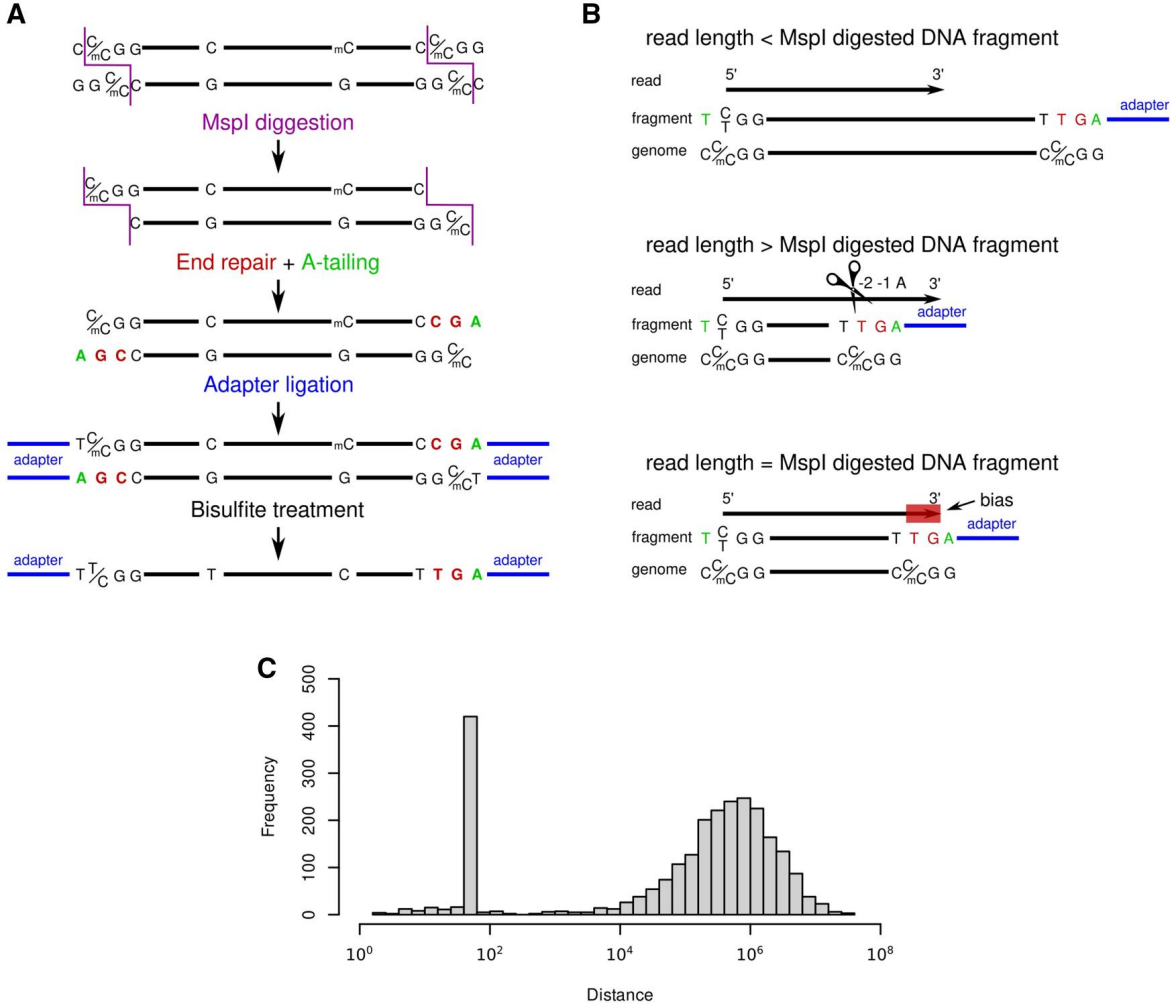
Graphical summary (A) Graphical representation of an RRBS read (black arrow) with 3′ end genomic MspI site overlap. Nucleotides attached to the fragment by end-repair are in red, A-tailing ligations are in green, adapters are in blue. Original genomic context is shown below. Biased methylation calling is due to the T in red. (B) The three potential contexts of read length and fragments length comparison. Trimming happens when the 3′ A is recognized by Trim Galore. In the absence of trimming the -2 biased nucleotide will be considered as non-methylated cytosine. (C) Distance distribution of neighboring DMSs before. Data from (Pagliaroli *et al.* 2021).

Reduced Representation Bisulfite Sequencing (RRBS) is a cost-effective method to analyze DNA methylation at single base resolution in CpG-rich regions at the genome-wide level (Meissner *et al.* 2005). The CpG-rich regions are obtained by endonucleolytic fragmentation followed by size-selection. Thus, the RRBS protocol begins with digestion of genomic DNA by MspI, which cleaves DNA at CCGG sites enriched in the CpG-rich regions. MspI is sensitive to the methylation of the 5′C, which is frequent in planta but extremely rare in animalia (Walder *et al.* 1983, Cokus *et al.* 2008). However, the enzyme is insensitive to the methylation of the internal CG. Therefore, RRBS can be used for methylation analysis of animal tissues without introducing bias. Several similar protocols exist and they all contain before or after the bisulfite treatment an end-repair step. At this step, a cytosine in CG position is introduced to the 3′ ends of the DNA fragments (Fig. 1A). Since almost only non-methylated cytosines are used, for simplicity we will focus on end-repair with non-modified cytosine in this manuscript. Bisulfite treatment converts all unmethylated cytosines to uracil, including those introduced by the end-repair. RRBS data analysis after NGS requires specialized tools. Commonly used computational pipelines utilize Trim Galore (Krueger 2015), a wrapper around CutAdapt (Martin 2011), and FastQC (Andrews 2010) for quality and adapter trimming of the RRBS reads. Since end-repair adds an unmethylated cytosine to the 3′ end of the fragments, it decreases the detected methylation level at the CG sites where they map.

Furthermore, these biased sites can be identified erroneously as differentially methylated sites (DMSs), thus, confounding the RRBS analysis results. Therefore, eliminating this additional cytosine is crucial to accurately determine methylation levels. For this purpose, Trim Galore is implemented with an ‘—rrbs’ option that trims the end-repaired cytosine when an adapter sequence is detected. However, if the adapter is not detected because the 3′ end of the read is exactly at the MspI site, the end-repaired cytosine will be considered in methylation calling. This was the case in a recent study, when we encountered an unexpectedly high number of DMS pairs 50 nucleotides apart when a single-ended (SE) RRBS was performed with 50 nucleotide sequence reads from rat striatum samples. The biased DMSs were manually curated, but the need for an automated solution emerged (Pagliaroli *et al.* 2021).

Here, we propose improve-RRBS, a sequencing length-independent, easy-to-use python package to improve the precision of RRBS methylation calling of both paired-end (PE) and SE sequencing data.

## 2 Methods

### 2.1 Improve-RRBS

The improve-RRBS python package can be smoothly implemented in common RRBS analysis pipelines. Here, we used Trim Galore (v0.6.10) (*n* = 1, ‘—rrbs’ and default parameters) (Krueger 2015), Bismark (default parameters) (v0.24.2) (Krueger and Andrews 2011), and methylKit (v1.28.0) (Akalin *et al.* 2012, Wreczycka *et al.* 2017). The improve-RRBS package is installed by pip by running ‘pip install iRRBS’. To run improve-RRBS the following input parameters are required in the subsequent order:

Infile (-i): path to input sorted BAM file, chromsizes (-c): path to chrom.sizes file to define the chromosome lengths for a given genome, genome (-g): path to genome file, outfile (-o): name for the output file. Improve-RRBS depends on SAMtools (Li *et al.* 2009), BEDTools (Quinlan and Hall 2010), and pysam (https://github.com/pysam-developers/pysam) and pybedtools (Dale *et al.* 2011).

Improve-RRBS identifies whether the reads are SE or PE. In case of PE, only R1 reads are processed since all R2 reads have been already trimmed by Trim Galore, if the ‘—rrbs’ option was used (Krueger 2015). The properties of the reads are identified by reading their flags. The unique mapping locations of the SE or PE R1 reads are converted into BED format. Only the unique mapping locations are kept to reduce calculation time. Then, these mapping locations are extended by two additional upstream and downstream bases. Subsequently, the genomic sequences of the unique mapping locations are extracted. If the 3′ end of the extended location overlaps with a genomic MspI site (CCGG) (Fig. 1B), that location is reduced back to the original size and marked as ‘MspI block’. The reads that exactly overlap with these blocks are extracted from the input BAM file. These reads are grouped and processed separately based on their strand orientation. The three characters of their XM tag that represents the methylation status of the MspI overlapping nucleotides are replaced with ‘not cytosine’ characters. As a result, the end-repair added cytosines are masked for methylation level calling to prevent bias. Datasets of the order of ten million reads can be processed within 10 minutes, even on a desktop computer with 4 cores and 16 GB RAM.

### 2.2 DMS and DMR identification

Differential methylation analysis was performed as in Pagliaroli *et al.* (2021). Briefly, the RRBS data was analyzed using MethylKit. Initially, only Cs with a minimum of 10 reads were considered for methylated and unmethylated cytosines in the CpG context. In case of the rat samples, a threshold of 10 reads per CpG was required in at least four out of six samples in each group. Only samples meeting this threshold were considered for further methylation analysis. Differentially methylated sites (DMSs) were then identified using the ‘calculateDiffMeth()’ function, which is a logistic regression-based method. The analysis was performed in two ways: with the default parameters and a more stringent analysis, with over-dispersion correction, as recommended by MethylKit's (Wreczycka *et al.* 2017) creators. Differentially methylated regions (DMRs) were also identified with MethylKit by using its tiling window method (window.size = 1000, step.size = 200). Only windows including at least 5 CpGs were analyzed. At least 20% methylation was required to identify a region as DMR. In both cases, the results were further filtered based on a significance threshold of *q* < 0.01.

### 2.3 TRACE-RRBS

TRACE-RRBS (version 0.1) (Baheti *et al.* 2016) was used with default settings unless modifications to the parameters were required to allow the execution of the analysis. For the *in silico* digestion of the genome, using the tool ‘methyl_fragment_builder.jar’, parameters were tailored to match the characteristics of the specific sequencing data. According to the user manual of the software, the maximum length of a fragment was set to 250. The minimum fragment length varied depending on the sequencing length: it was set to 30 for sequencing lengths of 50, 51, and 63 (after removal of Unique Molecular Identifier); to 50 for a sequencing length of 101; and to 100 for a sequencing length of 150.

## 3 Results and discussion

The bioinformatic analysis of RRBS results starts with the quality check of the raw data. This step is followed by trimming the adapters and low sequencing quality bases at the end of the reads. Trimming is a crucial step carried out by Trim Galore, which also cuts the unmethylated cytosines introduced by end-repair. The remaining end-repaired cytosines (read as thymines after the bisulfite conversion) would result in biased methylation calling. Trim Galore works adequately when the fragment size is shorter or longer than the sequencing read length (Fig. 1B). Indeed, Trim Galore recognizes the Adenine (and the following adapter) (Fig. 1B) and cuts the 3′ end of the read to avoid the inadequate evaluation of DNA methylation at the 3′ MspI site (TTGA after RRBS). When the read length is longer than the fragment size, the adapter is sequenced and easily recognized before cutting. When the read length is shorter than the fragment size, the end-repaired cytosine at the 3′ end of the fragment is not sequenced. We hypothesized that Trim Galore fails when the fragment and read sizes are of equal length; therefore, the A at the 3′ end of the fragment can not be detected. Hence, end-repaired cytosine of the 3′ MspI site would not be cut. We tested our hypothesis on several datasets from independent laboratories investigating different species (Supplementary Table 1, available at *Bioinformatics Advances* online). In all datasets tested, we found reads ending by 3′ TTG after trimming. A non-negligible portion of these reads had the same length as the sequencing length determined by the experimental setup. Hence, the 3′ end of these reads was not removed by Trim Galore, suggesting that some of these reads represent DNA fragments ending by a TTG overlapping with a genomic MspI site, where the middle T is artefactual. The fraction of reads containing 3′TTG triplets was between 1.72% and 3.33% for R1 reads or single-ended (SE) sequences (for R2 reads of paired-end sequencing, see below). We did not observe any correlation between read length and percentage of reads with untrimmed 3′TTG.

In the RRBS analysis pipeline, trimming is followed by mapping and then methylation calling is performed. If no trimming is performed at a MspI site at the 3′ end of a read, this leads to biased methylation calling. MspI site at the 5′ end of the read does not undergo end-repair. Therefore, there is no methylation bias (Fig. 1A). After methylation calling of all CG dinucleotides in a sample, differentially methylated sites (DMS) can be identified by comparing multiple samples. The results of the bias can be easily observed by determining the distance distribution of neighboring DMS. The artifact is demonstrated by the presence of a peak in the distance distribution. The peak location equals the sequencing read length (Fig. 1C).

**Figure 2.**
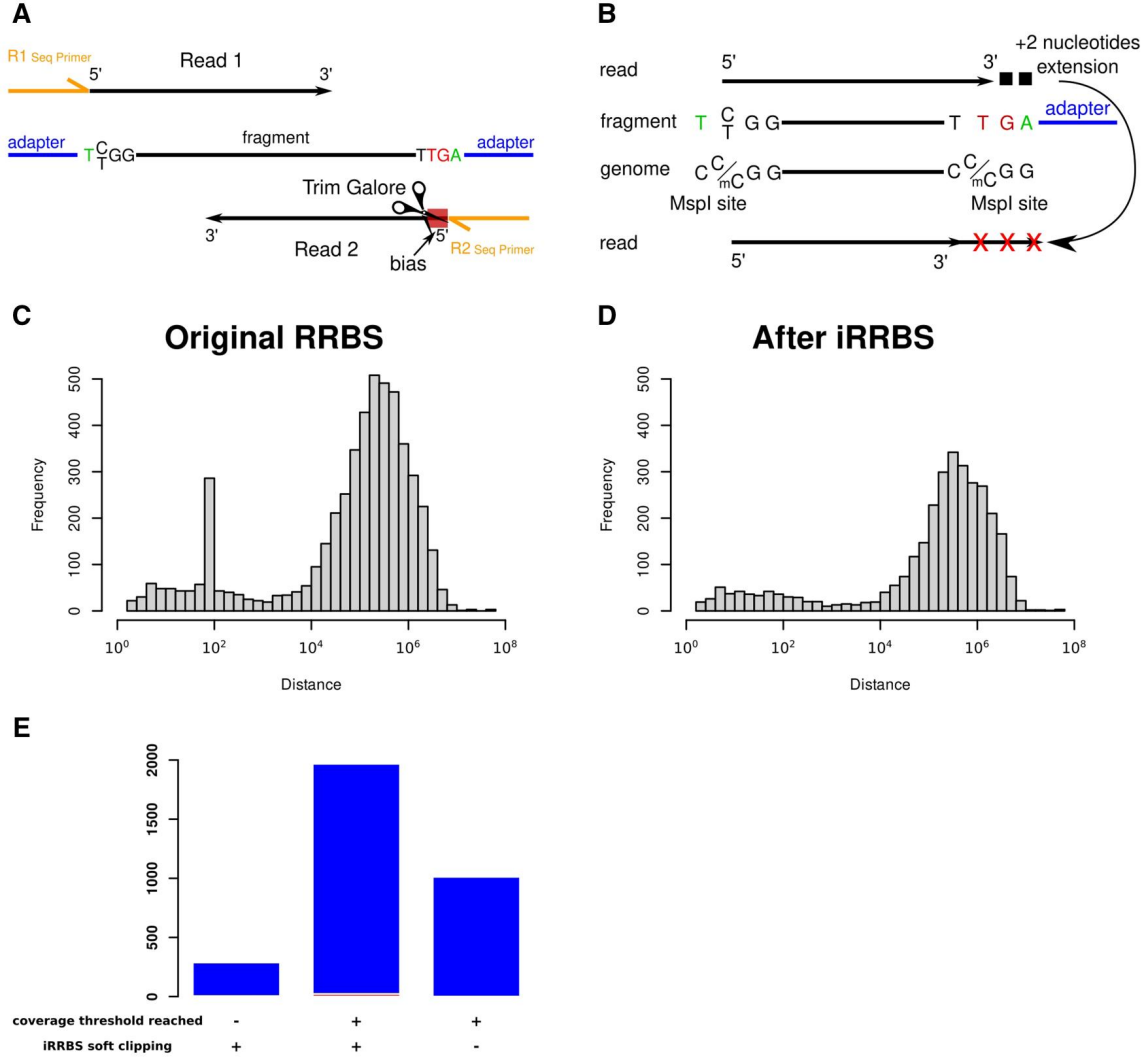
Mechanism and results of action of iRRBS. (A) No action on R2 reads is required, as R2 uses as template either the original top strand or original bottom strand and starts sequencing from the 3′ end of the fragment. Therefore, the 5′ end of the read is always biased since it is complementary to the endrepaired 3′ end of the original fragment. This part is trimmed by Trim Galore when the ‘—rrbs’ parameter is used. The 3′ end of the R2 read is complementary to the 5′ end of the original read, which is never biased. (B) Shows the mechanism of action of iRRBS. The software uses the mapped reads, adds two nucleotides from the genomic region (black squares) to the 3′end of the sequence and if this identifies an exact overlap with an MspI site, the last three nucleotides of the R1 read will be masked (shown by red X) for methylation analyses. Distance distribution of neighboring DMSs before (C) and after (D) iRRBS. Data analysis of RRBS sequenced with two different fragment length. (E) Distribution of lost DMS due to the use of iRRBS. Blue columns indicate the false positive DMS filtered out. True positive DMS lost due to the masking of MspI sites, which underwent false positive masking are indicated in red. Data from (Reizel *et al.* 2015, Stubbs *et al.* 2017).

To circumvent this problem, we developed improve-RRBS, which detects and avoids the inclusion of end-repaired cytosines in methylation analysis. In the modified pipeline, improve-RRBS is executed after mapping the quality filtered and trimmed reads (sorted BAM files) but before methylation calling (Supplementary Fig. 1, available at *Bioinformatics Advances* online). First, the tool identifies whether the reads are SE or PE. Next, the mapping locations of SE or R1 reads are extended by two additional upstream and downstream bases. In case of PE, only R1 reads are processed since, independently of their length, the 5′ end of R2 reads are biased, and Trim Galore trims them when the ‘—rrbs’ option is used (Fig. 2A). In contrast, R2 reads are not biased at their 3′ end as they are complementary to the original top strand or bottom strand (CTOT and CTOB, respectively), which are never biased at their 5′ end. Subsequently, if the 3′ end of an extended SE or R1 read overlaps with a genomic MspI site, the last three nucleotides of the 3′ end of the original read are masked for methylation calling by iRRBS to prevent bias (Fig. 2B).

Before proceeding with further investigations we assessed whether iRRBS also masks false positive CpGs. Using the 12 PE samples from earlier, we evaluated the specificity of our method. We defined a false positive as a fragment mapped to a genomic MspI site at the predefined read length, then masked, with another MspI site detected further 3′ on R2. To avoid artifacts the minimum required distance between the fragment and read length for this analysis was 4 bp, the length of an MspI site. This ‘false positive’ scenario, potentially masking ‘true’ CpGs instead of end-repaired ones, may be due to SNPs (impossible to detect in the setup) or closely spaced MspI sites. Our findings showed that only 0.25%–0.5% of soft-clipped reads were false positive (not shown), reassuring us to proceed with analyzing the usefulness of improve-RRBS.

To further test our method, we reanalyzed different datasets by a pipeline that utilizes Trim Galore, Bismark (Krueger and Andrews 2011), and methylKit (Akalin *et al.* 2012). In the new pipeline, improve-RRBS was implemented between Bismark and methylKit. We started the validation with the recent dataset from our lab (Pagliaroli *et al.* 2021) (Supplementary Table 1, available at *Bioinformatics Advances* online). Sequencing was performed by SE and 50 nucleotides read length. The novel improve-RRBS algorithm identified 1.04%–1.38% of the 7–22 million trimmed, quality-checked, and mapped reads to contain artificial cytosine. For that reason, when the traditional pipeline was used, an increased number of DMS pairs 50 nucleotides apart were detected. The closest downstream DMS was at precisely 50 nucleotides distance in 10.2% of all DMSs. Similar to manual curation, improve-RRBS significantly decreased the number of DMSs. However, improve-RRBS had an even more significant effect, and it eliminated almost 75% and 40% of the DMSs when using stringent (Wreczycka *et al.* 2017) or classic detection parameters (Akalin *et al.* 2012), respectively (Supplementary Table 2, available at *Bioinformatics Advances* online).

We continued by testing for the presence of artificial unmethylated cytosines in all datasets used in our initial analysis presented in Supplementary Table 1, available at *Bioinformatics Advances* online. We observed that all datasets contained biased, untrimmed cytosines, overlapping with genomic MspI sites at the 3′ ends of the reads.

Next, we investigated the influence of these untrimmed nucleotides on methylation calling. A PE dataset from mouse kidney samples with high read depth (average of 26 million mapped R1 reads) (Guan *et al.* 2020), and we identified ∼1.2% with untrimmed end-repaired confounding cytosine (Supplementary Table 1, available at *Bioinformatics Advances* online). However, these artifacts induced less than a 0.2% change in the number of DMSs (Supplementary Table 2, available at *Bioinformatics Advances* online). It should be noted that in this experiment we compared three control samples to three other controls randomly chosen from an original set of six controls. Therefore, the experimental conditions were identical causing the homogenous distribution of artefactual cytosines and the small percentage of biased DMS. Our results also suggest that the number of the confounding cytosines is not linearly correlated with the size of their effect, but they might depend on the wet-lab experimental conditions as well (e.g. the kit used, etc). It is noteworthy that within the same experiment the percentage of biased cytosines was always highly similar between samples (Supplementary Table 1, available at *Bioinformatics Advances* online).

We hypothesized that the untrimmed cytosines would cause the most substantial bias when the differential methylation analysis is performed between two datasets with different read lengths. We reasoned that different CGs would be biased in the two datasets since the position of the untrimmed end-repaired CGs depends on the sequencing read length. Our method was tested on two PE datasets from the liver of one-week-old male mice, one with 100 nucleotides long reads and the other with 75 (63 + Unique Molecular Identifier) (Reizel *et al.* 2015, Stubbs *et al.* 2017). While the samples with shorter reads had an average of 0.61% biased reads, 3.45% of the longer reads contained untrimmed artifacts (Supplementary Table 1, available at *Bioinformatics Advances* online). Almost 10% of the original DMSs were 100, 99, or 98 nucleotides away from their neighboring DMS (Fig. 2C). In this comparison another peak of neighboring DMS at 63 bp distance was initially expected. However, during library preparation Stubbs *et al.* (2017) selected fragments ∼100 bp and longer explaining the low percentages of biased reads (Supplementary Table 1, available at *Bioinformatics Advances* online) and the absence of the second peak on Fig. 2C. Masking the 3′ end of the biased reads decreased the number of the DMSs by 37% (or 18% with the less stringent parameters). Our approach eliminated the artifactual DMSs, and the corresponding peak in the DMS distance distribution disappeared (Fig. 2D).

We investigated the disappearance of 3134 DMS in the less stringent analysis after iRRBS masking (Fig. 2E). We categorized the lost DMS into three groups. The first group comprised CpGs with remaining coverage below 10 reads per sample after masking, which were excluded from further analysis based on Methylkit parameters. The second group of CpGs underwent both masking and differential methylation analysis, but no significant differences were detected. This constituted the largest group. A very small number of DMS disappeared due to the false positive masking by iRRBS (indicated by red). The third group of CpGs was unaffected by iRRBS masking, yet the statistical method employed by Methylkit for controlling the false discovery rate resulted in the exclusion of these dinucleotides.

To demonstrate that the biased cytosines may cause the detection of false positive DMS in all sample set and to highlight the effect of the comparison of samples with different read length, we compared the mouse datasets 1 and 4 (Reizel *et al.* 2015, Guan *et al.* 2020) (Supplementary Table 1, available at *Bioinformatics Advances* online). They have different read lengths and are originating from liver and kidney, respectively leading to 77125 DMS. However, improve-RRBS identified 1905 false positive DMS. As expected, the distance distribution analysis of the DMS with and without improve-RRBS identified a large proportion false positive DMS at exactly 100 and 150 bp (Supplementary Fig. 2, available at *Bioinformatics Advances* online), respectively, which equals the read lengths in the two experiments.

In addition to DMS, differentially methylated regions (DMR) are frequently identified in studies investigating DNA methylation. It has been shown that neighboring CpGs in the same genomic region have similar DNA methylation levels; therefore, identifying DMRs can give more valuable information about methylation differences between two datasets than individual DMS. Thus, we tested the datasets with or without the novel improve-RRBS tool for DMRs. We observed an infinitesimal change in the number of regions tested for differential methylation. However, we found a 78.25%, −0.07%, and 9.4% loss of DMRs in the comparisons we carried out earlier to detect the DMS demonstrating the usefulness of the novel tool (Supplementary Table 2, available at *Bioinformatics Advances* online).

**Figure 3.**
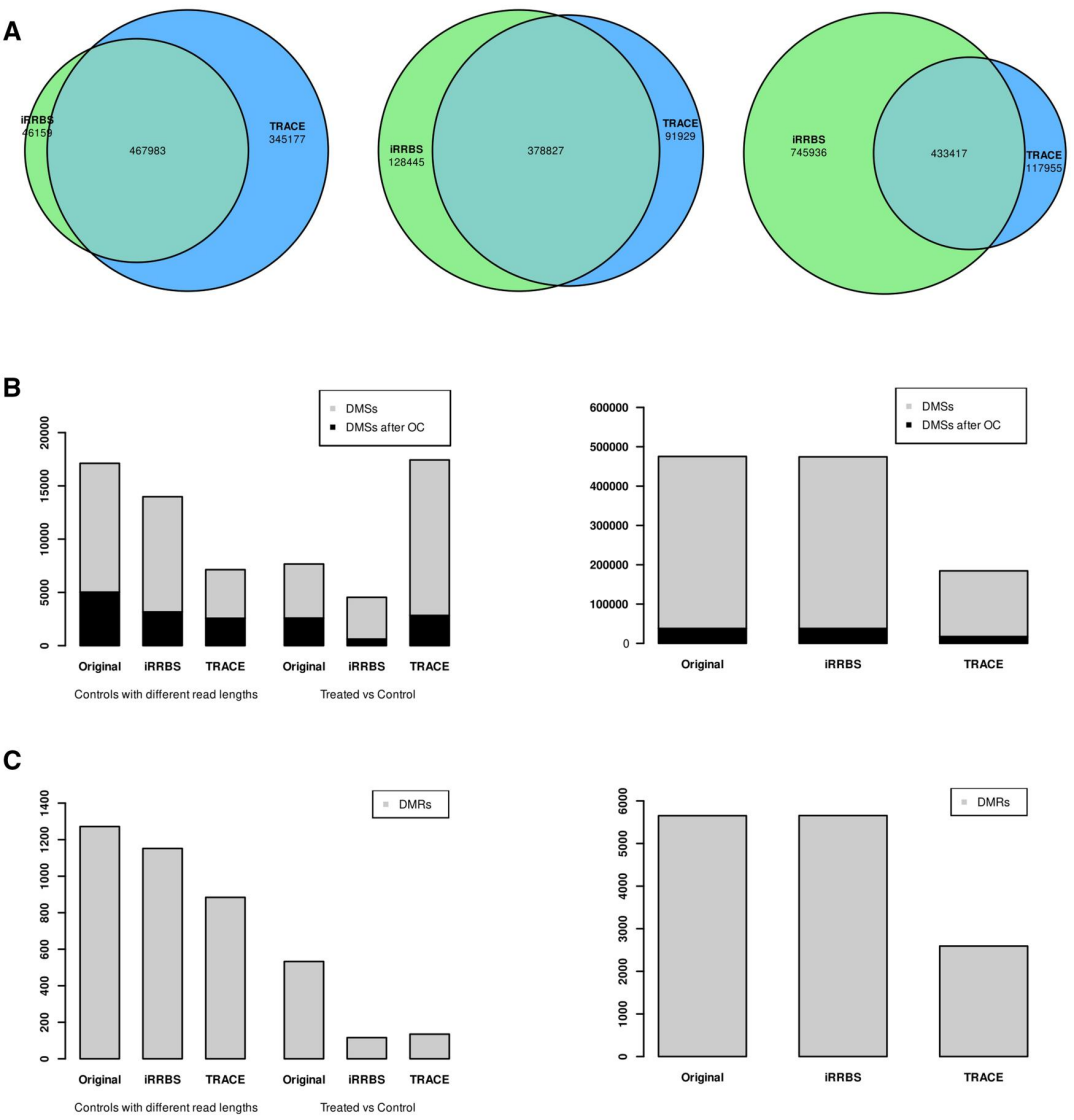
Comparison of improve-RRBS and TRACE-RRBS. (A) Venn diagram of CpGs identified prior differential methylation calculation (background). From left to right: 50 b SE sequencing (rat striatum); 75 b and 100 b PE sequencing (mouse liver); 150 b PE sequencing (mouse kidney). (B) DMS calculation before and after improve-RRBS (iRRBS) or TRACE-RRBS (TRACE) with (black) or without (grey) the more stringent overdispersion correction. Right panel indicates DMS from the kidney samples put separately to indicate the difference of scale. (C) DMR calculation on the same samples in the same order as in (B). Exact numbers are indicated in Supplementary Table 2, available at *Bioinformatics Advances* online.

Eventually, we compared improve-RRBS with TRACE-RRBS, a method proposed by Baheti *et al.* (2016). TRACE-RRBS involves a virtual MspI digestion of the genome followed by size-selection, creating a virtual genome for mapping RRBS reads. This approach offers an alternative solution to improve-RRBS to avoid the artefactual CpGs. We conducted analyses using TRACE-RRBS on the samples investigated earlier (Supplementary Table 2, available at *Bioinformatics Advances* online). Default parameters were used, with modifications according to read lengths. Initially, we compared the overlap between CpGs obtained for each comparison after mapping (Fig. 3A). For rat SE reads (50 b), we observed a ∼55% overlap, with 40% of CpGs identified only by TRACE-RRBS. However, with increasing read length to the commonly used 150 b PE, the situation changed drastically. With 100 b and 63 b comparisons, a 63% CpG overlap was detected, with significantly more CpGs detected after mapping by iRRBS than by TRACE-RRBS. In kidney samples sequenced with 150 b PE, 57% of CpGs were detected only by iRRBS, while <10% were TRACE-RRBS-specific.

Next, we performed DMS analysis using Methylkit on the datasets prepared by TRACE-RRBS and compared the results with those obtained by iRRBS (Fig. 3B). While TRACE-RRBS detected more DMS with 50 b SE samples than iRRBS, the situation reversed with the currently used 150 b read length, where improve-RRBS detected over twice as many DMS. Interestingly, when comparing DMRs, TRACE-RRBS lost its advantage with short reads but maintained a considerable disadvantage with longer reads (Fig. 3C).

## 4 Conclusions

The 3′ end-repaired cytosines can escape trimming by commonly used RRBS computational pipelines if the 3′ end of the read overlaps with the MspI site. This can alter methylation calling and sometimes produces a high number of false positive DMSs or DMRs. improve-RRBS eliminates these artificial cytosines and improves the accuracy of methylation calling. A python package has been developed to facilitate the implementation of the code.

## Supplementary Material

vbae076_Supplementary_Data

## Data Availability

Improve-RRBS is available at the pypi repository (https://pypi.org/project/iRRBS/), and all source codes are freely available at https://github.com/fothia/improve-RRBS.
