# Supplementary material for: Improve-RRBS: a novel tool to correct the 3′ trimming of reduced representation sequencing reads
Source: Bioinform Adv. 2024 May 24;4(1):vbae076. doi: 10.1093/bioadv/vbae076 (PMC11154647; doi:10.1093/bioadv/vbae076)
Supplement: vbae076_Supplementary_Data [file vbae076_supplementary_data.pdf]

Supplementary table 1.

Dataset	GEO Accession	Organism	Read length	Number of R1 or SE reads	Number of R1 or SE reads with TTG end	TTG/R1 or SE reads	Number of msp1 reads	Msp1/R1
1	GSM1463690	Mus musculus	101	12219254	279743	2.29%	429114	3.51%
	GSM1463691	Mus musculus	101	8698805	201662	2.32%	311340	3.58%
	GSM1463692	Mus musculus	101	9986447	222500	2.23%	327569	3.28%
2	GSM2465649	Mus musculus	63*	7885344	257552	3.27%	52694	0.67%
	GSM2465652	Mus musculus	63*	14955558	496776	3.32%	84922	0.57%
	GSM2465667	Mus musculus	63*	14139636	470554	3.33%	82935	0.59%
3	GSM5352558	Rattus norvegicus	50	17973232	528269	2.94%	248904	1.38%
	GSM5352559	Rattus norvegicus	50	15645217	455382	2.91%	204300	1.31%
	GSM5352560	Rattus norvegicus	50	20204935	596017	2.95%	270386	1.34%
	GSM5352561	Rattus norvegicus	50	22710110	666093	2.93%	280811	1.24%
	GSM5352562	Rattus norvegicus	50	13885541	406430	2.93%	184214	1.33%
	GSM5352563	Rattus norvegicus	50	15319800	447264	2.92%	204762	1.34%
	GSM5352564	Rattus norvegicus	51	10707085	319657	2.99%	128313	1.20%
	GSM5352565	Rattus norvegicus	51	7257697	212572	2.93%	80668	1.11%
	GSM5352566	Rattus norvegicus	51	11531005	348063	3.02%	163472	1.42%
	GSM5352567	Rattus norvegicus	51	10323483	306260	2.97%	107653	1.04%
	GSM5352568	Rattus norvegicus	51	7327033	216255	2.95%	82733	1.13%
	GSM5352569	Rattus norvegicus	51	8226596	242833	2.95%	94476	1.15%
4	GSM3940765	Mus musculus	150	44325455	840055	1.90%	542280	1.22%
	GSM3940766	Mus musculus	150	29290297	540373	1.84%	363215	1.24%
	GSM3940767	Mus musculus	150	27898798	517980	1.86%	344700	1.24%
	GSM3940768	Mus musculus	150	20948107	353933	1.69%	257218	1.23%
	GSM3940769	Mus musculus	150	24025222	413899	1.72%	291308	1.21%
	GSM3940770	Mus musculus	150	17841306	312795	1.75%	186627	1.05%

*After removing unique molecular identifier

RRBS statistics from different experiments revealing the overlap of R1 or SE reads with 3' MspI sites. RRBS samples tested were obtained from previously published studies (1-4, respectively) (Reizel, et al., 2015; Stubbs, et al., 2017; Pagliaroli, et al., 2021; Guan, et al., 2020)

Supplementary table 2

Comparison	Method used	Number of CpG sites (2024)	TRACE	DMSs with over-dispersion correction (2024)	TRACE	DMSs without over-dispersion correction (2024)	TRACE	Number of tiles (2024)	TRACE	DMRs	TRACE
Controls with different read lengths (1,2)	classical workflow	508159		5028		17117		224903		1272	
	iRRBS	507272	470756	3171	2577	13983	7134	224406	208126	1152	884
	% relative to classical workflow	99.83%	92.6%%	63.07%	51.25%	81.69%	41.67%	99.78%	92.54%	90.57%	69.50%
Treated vs Control (3)	classical workflow	516667		2592		7665		166771		533	
	iRRBS	514142	813160	622	2838	4535	17434	165437	277120	116	135
	% relative to classical workflow	99.51%	157.39%	24.00%	109.50%	59.17%	227.45%	99.20%	166.17%	21.76%	25.33%
Control vs Control (4)	classical workflow	1181523		38133		475263		479776		5652	
	iRRBS	1179353	551372	38077	17501	474340	184503	478406	237002	5656	2592
	% relative to classical workflow	99.82%	46.66%	99.85%	45.89%	99.81%	38.82%	99.71%	49.40%	100.07%	45.86%

Statistics of DMS and DMR identified with or without iRRBS or TRACE-RRBS. Same samples were used as in Supplementary Table 1.

Supplementary Figure 1

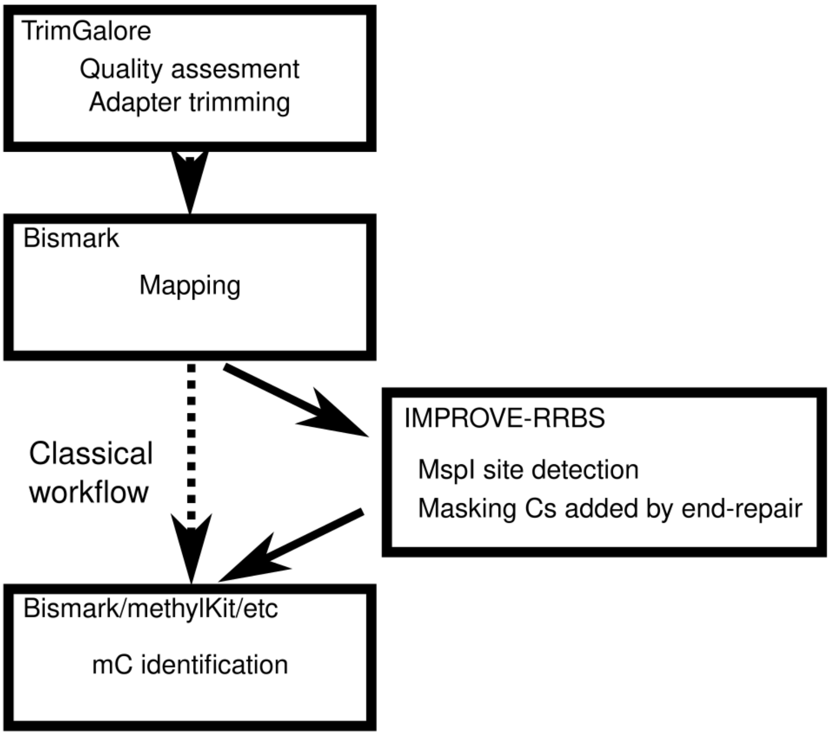

RRBS analysis pipeline. Classical pipeline on the left part of the figure. Proposed inclusion of iRRBS on the right side.

Supplementary Figure 2

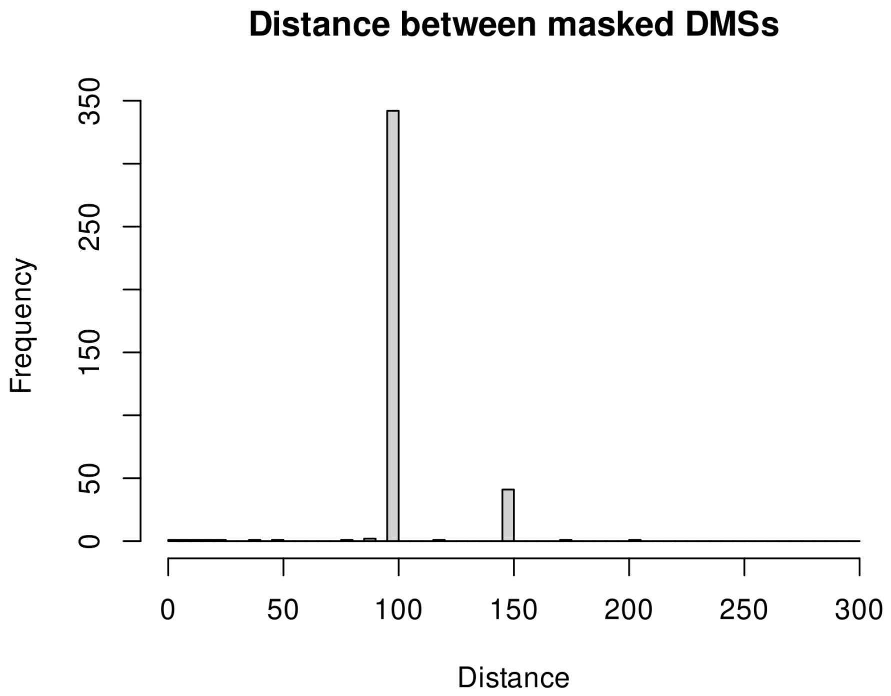

Distance distribution difference of neighboring DMS identified between dataset 1 and 4 with or without iRRBS tool. Distances below 300bp are shown.
